# Decreasing trend in severe weather occurrence over China during the past 50 years

**DOI:** 10.1038/srep42310

**Published:** 2017-02-17

**Authors:** Qinghong Zhang, Xiang Ni, Fuqing Zhang

**Affiliations:** 1 Department of Atmospheric and Oceanic Sciences, School of Physics, Peking University, Beijing 100871, China; 2 Department of Meteorology, and Center for Advanced Data Assimilation and Predictability Techniques, The Pennsylvania State University, University Park, Pennsylvania, United States

## Abstract

Understanding the trend of localized severe weather under the changing climate is of great significance but remains challenging which is at least partially due to the lack of persistent and homogeneous severe weather observations at climate scales while the detailed physical processes of severe weather cannot be resolved in global climate models. Based on continuous and coherent severe weather reports from over 500 manned stations, for the first time, this study shows a significant decreasing trend in severe weather occurrence across China during the past five decades. The total number of severe weather days that have either thunderstorm, hail and/or damaging wind decrease about 50% from 1961 to 2010. It is further shown that the reduction in severe weather occurrences correlates strongly with the weakening of East Asian summer monsoon which is the primary source of moisture and dynamic forcing conducive for warm-season severe weather over China.

Severe weather in general, refers to any destructive storm, but is “usually applied to severe local storms in particular, that is, intense thunderstorms, hailstorms, and tornadoes” (the online Glossary by the American Meteorological Society). Changes in local severe weather events under the warming climate are of great concern to the society^1^,^2^,^3^ but there are great challenges in understanding the linkage and correlations between the local weather variations and the global climate change. The difficulty is at least in part because severe weather often occurs in spatial scales within tens of kilometers and periods within hours, which is in stark contrast with the scales of the global climate change that are in thousands of kilometers and over decades or longer. Moreover, inhomogeneities and inadequacies in the monitoring systems, including the lack of reliable and coherent spatiotemporal coverages, present even greater challenges to understand trends in severe weather under the changing climate background^3^,^4^.

Nevertheless, limited progress has been made in developing and understanding severe weather climatology and trends using station observations, public records and/or damage reports^5^,^6^,^7^,^8^,^9^,^10^,^11^,^12^,^13^,^14^. There are also studies in using severe weather indices derived from global reanalysis and/or climate model simulations as proxies to understand the changes in the large-scale environment which is conducive to severe weather^15^,^16^. These indices include but not limited to the convective available potential energy (CAPE), vertical wind shear, atmospheric instability and strength of synoptic forcing mechanisms^17^. To the best of our knowledge, this study presents by far the most comprehensive analysis of severe weather trends based on continuous and coherent severe weather reports from 580 manned stations all across China during the past 5 decades. Also analyzed are the corresponding changes in the large-scale environment and severe weather indices derived from the global reanalysis dataset. We have selected 580 manned stations all across China that have continuous minutely-to-daily records of severe weather events from 1961 to 2010 with each station having missing report days less than 1% of the total days over the study period. All these stations are dedicated as the climatological observing stations certified by the World Meteorological Organization (WMO). These records are based on observations provided by professionally-trained meteorological observers and the data are archived at the China Meteorological Administration (CMA)^18^. The observational methodology of each type of severe weather events is provided in the Method section below and has been rather persistent over the study period. Some of these records have also been studied by other researchers from different perspectives^19^.

## Results

**Figure 1:**
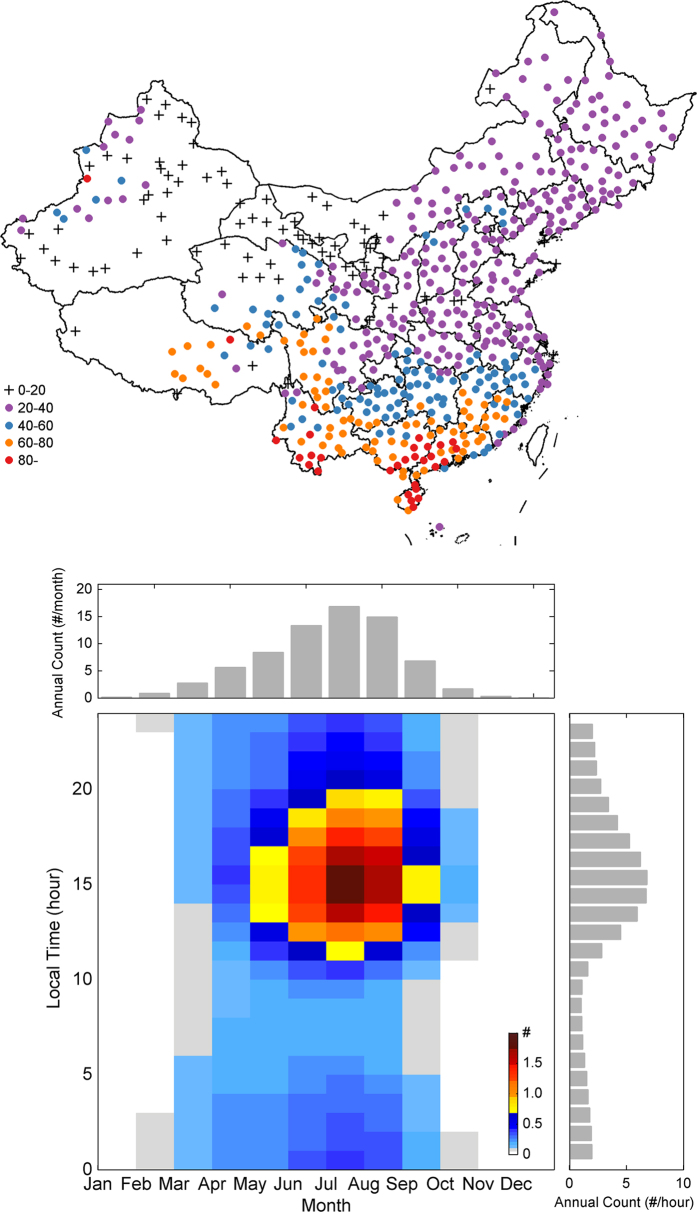
The spatial distribution of the annual mean thunderstorm days (top) and annual and diurnal variations of the mean number of thunderstorm number (bottom). The day and event numbers are averaged from 1961 to 2010. The annual mean thunderstorm numbers calculated by counting the time record number in the severe weather reports are counted at different times of the day and different months of the year shown on side bars. The figure is generated using MATLAB 2014b.

We first examine the frequency of occurrence and trends in thunderstorms across China during the past 5 decades (1961–2010). Figure 1a plots the color-coded annual-mean numbers of thunderstorm days at each of the 580 stations. It clearly shows that South China is the most-prolific thunderstorm-producing region with some stations reporting an average of more than 80 days per year. The frequency of thunderstorm days decreases gradually toward higher latitudes to less than 40 days per year in northern China. There is a secondary enhancement of thunderstorm occurrence on the east and southeast side of the Tibetan Plateau. Figure 1b shows the annual-mean distribution of the number of thunderstorm events at different months of the year versus different local time of the day that directly relates the thunderstorm occurrence to the solar heating annual and diurnal cycles. The thunderstorm season in China starts in early spring, peaks in the summer and ends in autumn with 84% of the total events occurred over the warm season months from May to September, during which most areas of China are strongly influenced by the East Asia summer monsoon^20^. Not surprisingly, most of the thunderstorms events occur during the local afternoon hours (from noon to before sunset) right after the peak of the short-wave radiation on the daily cycle.

**Figure 2:**
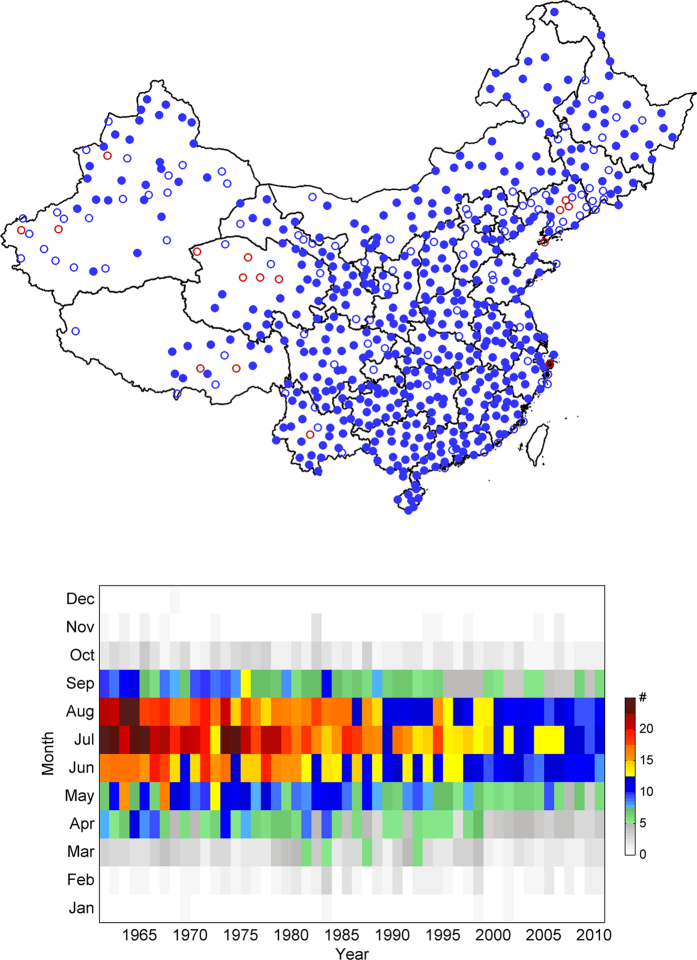
Station trends of thunderstorm days (top) and yearly evolution of thunderstorm events at each month (bottom). The blue (red) circle represents a decreasing (increasing) trend and trends above the 0.05 significance level are marked with filled circles. The figure is generated using MATLAB 2014b.

Figure 2a shows that the annual-mean number of thunderstorm events has a decreasing trend at an overwhelming majority of the 580 stations across China during 1961–2010, with most stations experiencing a decreasing rate of around 1% per year east of the Tibetan Plateau. The large decreasing trend also occurs over almost every month but in particular during the peak season from May to September (Fig. 2b); these warms-season months account for 84% of the annual mean total number of thunderstorm events.

**Figure 3:**
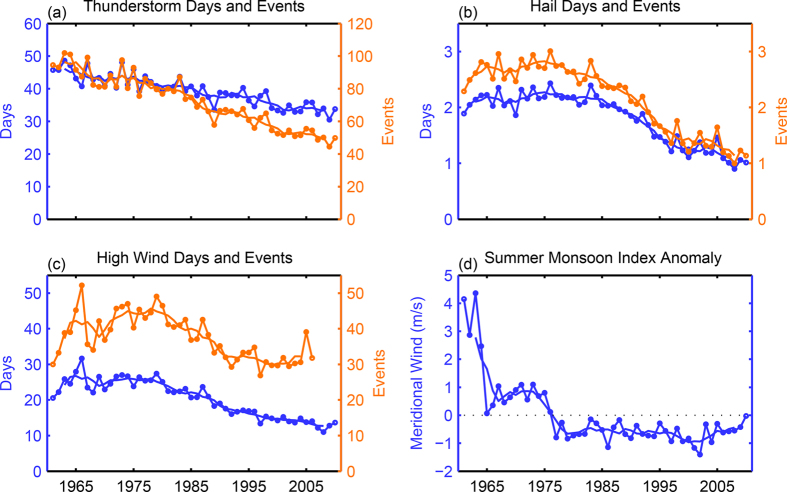
Yearly evolutions of severe weather days and events (**a–c**) and the East Asian summer monsoon anomaly (**d**). The station-averaged numbers of severe weather days and events are in blue and orange, respectively. The 5-year running mean (solid lines) is shown in each panel along with the unsmoothed original curves (with dots). The East Asian summer monsoon index is calculated as the mean anomaly of the 850-hPa meridional wind over (20.0°–40.5°N, 105.0°–120.0°E) in the warm-season months from May to September each year from 1961 to 2010.

The persistent decreasing trend in thunderstorm occurrence over China during 1961–2010 can be more clearly seen in Fig. 3a, which shows the time evolution of the annual-/station-mean number of thunderstorm days and number of events averaged over all 580 stations. The average number of thunderstorm days per station per year decreases from around 45 in 1960 s to about 34 days in 2010 with a linear decreasing trend of 2.82 days per decade. In the meantime, the average number of thunderstorm events per station per year decreases from around 100 in the early 1960 s to less than half of that total by the end of the study period with a linear decreasing trend of more than 10 events per station per decade. As also shown in Fig. 2, this decreasing trend is also reported in almost every sub-region of China throughout the year.

Coincident with the decreasing trend in the frequency of thunderstorm events, the occurrence frequency of all other reported local severe weather indicators also decreases drastically during 1961–2010. Figure 3b,c show the time evolution of the annual- and station-mean numbers of hail events and days, and damage wind event days, both of which have also experienced a significant decreasing trend over this period in China. While the station and annual mean hail days and number of hail events in China shows less persistent trend during the first part of the reporting period (1961‒1980), these numbers drop to less than half of the early average by early 2000 s (Fig. 3b). The number of high wind events and the number of high wind days both drop sharply with the annual and station mean numbers dropped to less than 40% over the study period (Fig. 3c). The numbers of reported lightning and tornado days are also decreasing but we choose not to show their trends given the likely changes in the detecting and reporting methodology of these events.

The drastic reduction in the severe weather occurrences over China during the past five decades coincides with a fast warming global climate along with rapid, multi-dimensional environmental changes (such as air pollution, land use and land cover) which are at least partly induced by rapid socioeconomical development, urbanization, and population growth. It is beyond the scope of the current study to pinpoint the complex mechanisms that may have caused the significant decreasing trends in the severe weather occurrence over China during this 50-year study period.

Nevertheless, given most of the local severe weather events occurred over the warm season months when most areas of China are strongly influenced by the East Asia summer monsoon, it is natural for us to explore whether the changes in severe weather are related to the changes in the large-scale monsoon circulations (while keeping in mind that correlation does not necessarily lead to causality). We examine a widely used summer monsoon index^21^ that is based on the strength and direction of the annual mean low-level (850 hPa) meridional wind averaged over (105.0°–120.0°E, 20.0°‒40.5°N) during the warm-season months from May to September as shown in Fig. 3d. The stronger and the more positive is the mean meridional wind, the stronger the East Asian summer monsoon since a more southerly wind can bring warmer and moister air from the south. Although the variations in this annual-mean monsoon index (Fig. 3d) appear to be more nonlinear and episodic (than the severe weather indicators shown in Fig. 3a–c), there is also an overall sharp reduction in the strength of this index during 1961‒2010 which calculated with the NCAR-NCEP global reanalysis dataset^22^. The mean meridional wind changed from around 2–4 m/s southerly in the early 1960 s to around 1 m/s between 1965 and 1975 but evolved into the negative (northerly) regime thereafter. The lowest value of this monsoon index occurred during early 2000 s (with the mean meridional wind anomaly of around −1 m/s). Weakening of the Asian summer monsoon has also been previously reported using both global reanalysis and simulations^23^,^24^,^25^.

**Figure 4:**
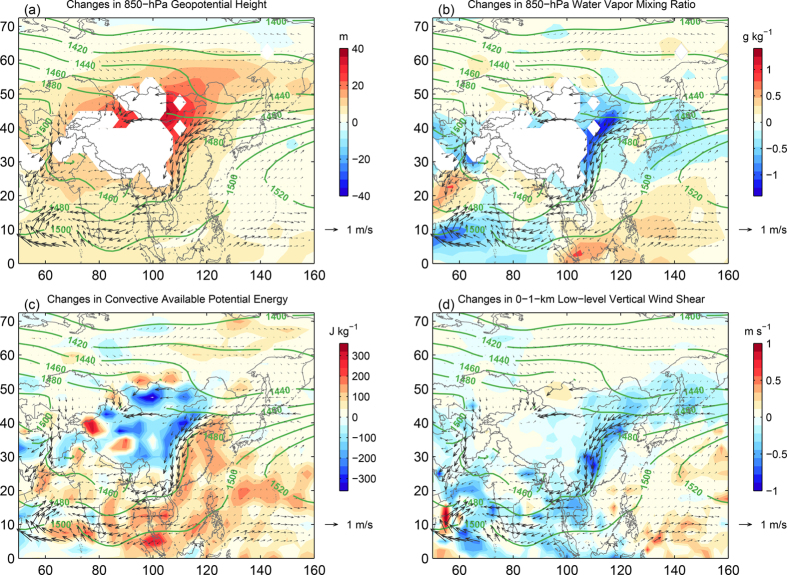
The differences of large-scale atmospheric environmental conditions. The results are the differences of the second 25-year average (1986–2010) and the first 25 year (1961–1985) for the (**a**) 850-hPa geopotential height, (**b**) 850-hPa water vapor mixing ratio, (**c**) convective available potential energy (CAPE), and (**d**) the low-level (0–1 km) vertical shear averaged over the warm-season months from May to September. In each panel for each comparison and reference, the 25-year average of the 850-hPa geopotential heights during 1986–2010 and the difference in the 850-hPa wind vectors between the two periods are overlapped in contour lines and arrows, respectively. The CAPE is calculated using default function from the NCAR Command Language (NCL) software package^26^. The figure is generated using MATLAB 2014b.

Motivated by the apparent positive correlations between the changes in local severe weather occurrence and the change in the East Asian summer monsoon index, we further examine the mean changes in the large-scale circulation and thermodynamic environmental conditions during the warm season derived from the same global reanalysis dataset. Figure 4 shows the changes in the mean 850-hPa geopotential height, 850-hPa water vapor mixing ratio, column-maximum convective available potential energy (CAPE), and 0–1-km low-level vertical wind shear from the first half to the second half of the study period (mean of 1986–2010 minus mean of 1961–1985), respectively; for easy comparison and reference, superposed on each panel are the mean changes in the 850-hPa horizontal wind vectors and the mean 850-hpa geopotential height during the second half of the study period (1986–2010).

The mean geopotential height at 850-hPa show a typical large-scale pattern of the summertime East Asian low-level trough to the leeside of the Tibetan Plateau which extends from Northeast to South China. As part of the East Asian summer monsoon, this synoptic trough system brings warm moist air from the south, which is the breeding ground for severe weather for most areas of China. However, there appears to have large changes in the strength of this large-scale low-level trough from the first 25 years to the second 25 years of the study period (1961–2010) as estimated from the NCEP/NCAR global reanalysis (Fig. 4a): almost all areas in this trough region have experienced a positive 850-hPa geopotential tendency suggesting a broad-based filling and weakening of the synoptic trough system all across China. This weakening is typified by the changes in the low-level horizontal wind vectors also shown in each panel; the southeast low-level jet to the east of the trough is seen to be reduced by as much as 5–7 m/s, which is a strong indication of a weakened monsoon flow from the first to the second half of the study period. Corresponding to the weakening synoptic trough is the reduction of the low-level moisture over broad areas in particular with the most reduction in the low-level southwesterly jet to the east of the synoptic trough (Fig. 4b). Consistent with changes in the low-level monsoonal trough, jet and moisture, and more pertinent to productivity of localized severe weather, there are also considerable reductions over broad areas of China in the moist instability (indicated by CAPE in Fig. 4c) and dynamic forcing (indicted by the 0–1-km low-level vertical wind shear in Fig. 4d) from the first half (1961–1985) to the second half (1986–2010) of the study period.

**Figure 5:**
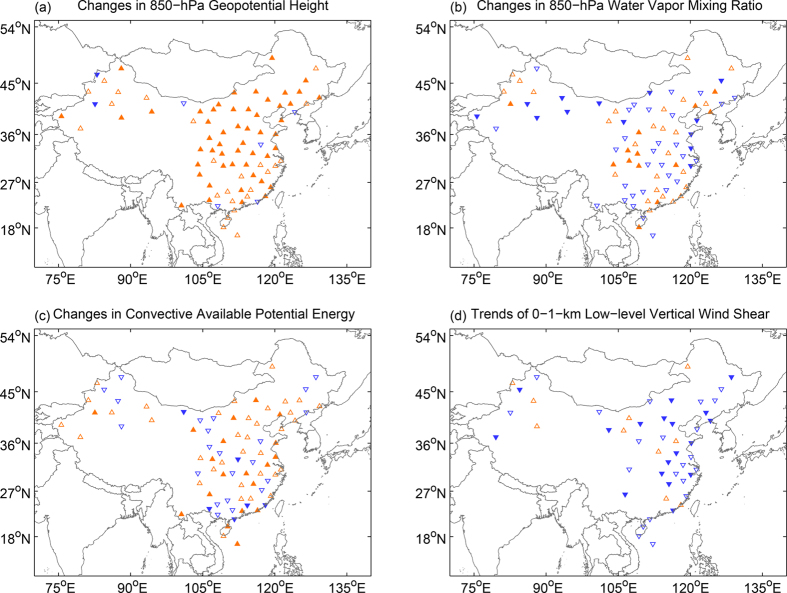
The trend of parameters derived from station sounding. (**a**) 850-hPa geopotential height from 1961 to 2010, (**b**) 850-hPa water vapor mixing ratio from 1961 to 2010, (**c**) convective available potential energy (CAPE) from 1961 to 2010. (**d**) low level (0–1 km) vertical shear from 1980 to 2010. Stations with significant trends are in filled markers. The figure is generated using MATLAB 2014b.

The positive 850-hPa geopotential tendency, reduction of moisture could also be directly inferred from station sounding observations (Fig. 5). Except for three stations that have insignificant downward trends, all other stations in East and North China show an increase in 850-hPa geopotential tendency (Figure 5a). Meanwhile, there are also more stations that have decreasing trend of 850-hpa moisture that with those with an increasing trend (Fig. 5b). Correspondingly, both the CAPE and vertical wind shear tendencies calculated from the sounding observations (Fig. 5c,d) are consistent with tendencies derived from the analysis from NCEP/NCAR reanalysis data (Fig. 4c,d).

Understanding the changes of extreme weathers frequency under global warming situation is a challenge for scientist. The challenge comes from our incomplete knowledge of complex physics processes and the lack of reliable and persistent long-term observations of small-scale moist convection. CMA of China begins to report and archive various surface weather phenomenon observations since around 1950, which is a valuable resource for examining and understanding severe weather trends. We chose 580 stations over the past 5 decades (from 1961 to 2010) to ensure a relatively large and continuous data record with a low data missing rate in this study. The analysis shows the numbers of thunderstorm and damage wind days have a steady decreasing trend from 1961 to 2010, with rates of 2.82 and 3.18 days per decade, respectively. The number of hail days on the other hand has rather small changes before 1980 s but decreases very sharply with a rate of 0.45 days per decade after 1980 s. Since an overwhelming majority of the severe weather events occurred in the warm season, most of the reduction in thunderstorm days also comes from May to September. Although correlation does not necessarily mean causality, we find that the decrease in severe convective weathers over China is strongly related to the weakening Asia summer monsoon that is associated with the weakening of dynamic forcing and the decreasing of moisture supply in the warm season. Nevertheless, it is beyond the scope of the current study to pinpoint the exact physical mechanisms that are responsible for such a rapid decrease of occurrence in the severe convective weather events. The possible contribution of a weaker summer monsoon to the decreased convective days in China warrants more research in the future, along with other factors in a more global perspectives.

## Data and Method

The severe weather reports used here were obtained from the Chinese National Meteorology Information Center (CNMIC), which has a complete historical surface weather phenomenon data set of 983 stations over mainland China from 1951 to present. As documented in the official reporting guidelines of CMA^18^, the report data contains surface-based observations of various weather phenomena, including but not limited to, snow, rain, fog, mist, haze, dust, hail, thunder, lightning, and tornado, along with each of the occurrence time. Thunder is recorded when cumulonimbus clouds are seen and thunders are heard by the professionally trained observers on duty of the station. We chose to focus on the thunder, hail and damage wind events to represent the severe convective weathers. Among them, both hail and thunder events have records of the starting and ending times down to the minute in the whole study period and the damage high wind’s time record terminated since 2007. If the time interval between two thunders is less than 15 minute, only this will be recorded as one event; otherwise, they are considered as two different events. If only one thunder is heard, only the starting time is recorded. We choose the starting time as the occurrence time of each thunderstorm (hail) events. The day record starts at 2000 BJT (Beijing Time, which is 1200 UTC) of the previous day per CMA recording convention^18^. If a thunderstorm starts before 2000 BJT, it belongs to the calendar day recorded. If a thunderstorm starts on or after 2000 BJT, it will be recorded to the next day. If a thunderstorm, hailstorm or damage wind event is recorded at one station on a particular day, we define it as an event day for this station. Relocated stations and stations with data missing rate greater than 1% are excluded. Finally, 580 stations with complete records from 1961–2011 were selected to ensure a relatively large and continuous data record (Fig. 1a).

The NCEP/NCAR reanalysis data and atmospheric sounding observations are used to analyze the changes of large scale atmosphere circulation in this study^22^. The reanalysis data are provided by the National Center for Atmospheric Research (NCAR). The atmospheric sounding observations from 146 weather stations are obtained from CNMIC but only 104 stations started observation from 1961. In our analysis, if there are more than 20% of observations missing in the warm season of a year, the record of that year is regarded as an unusable year; if there are more than 10 unusable years for one station during our study period, the record of that station will be excluded in the analysis of this study. Relocated stations are also excluded in this study. Each station has sounding data twice daily at 0800 LT (local time, 0000 UTC) and 2000 LT (1200 UTC), respectively. Considering most severe storm occurred in afternoon, we only use sounding observation at 0800 LT that are more representative of the large-scale environment. Since the wind observations in the sounding database we have access to now are available from 1980, only the variation of the mean vertical wind shear from 1980 to 2010 is shown in Fig. 5.

## Additional Information

**How to cite this article**: Zhang, Q. *et al*. Decreasing trend in severe weather occurrence over China during the past 50 years. *Sci. Rep.* **7**, 42310; doi: 10.1038/srep42310 (2017).

**Publisher's note:** Springer Nature remains neutral with regard to jurisdictional claims in published maps and institutional affiliations.
